# Insufficient lymph node assessment in gastric adenocarcinoma

**DOI:** 10.1186/s43046-019-0004-1

**Published:** 2019-10-22

**Authors:** Nezhat Khanjani, Sepideh Mirzaei, Hamid Nasrolahi, Seyed Hasan Hamedi, Ahmad Mosalaei, Shapour Omidvari, Niloofar Ahmadloo, Mansour Ansari, Fatemeh Sobhani, Mohammad Mohammadianpanah

**Affiliations:** 1 Department of Radiation Oncology, Shiraz University of Medical Sciences, Shiraz, Iran; 2 Shiraz Institute for Cancer Research, Medical School, Shiraz University of Medical Sciences, Shiraz, Iran; 3 Breast Diseases Research Center, Shiraz University of Medical Sciences, Shiraz, Iran; 4 Colorectal Research Center, Shiraz University of Medical Sciences, Shiraz, 71936 Iran

**Keywords:** Gastric cancer, Adenocarcinoma, Stomach, Lymph node dissection, Surgery

## Abstract

**Background:**

This study aimed to investigate the sufficient (≥ 16) lymph node assessment in 449 patients with gastric adenocarcinoma and literature review.

**Methods:**

Four hundred and forty-nine patients with pathologically confirmed locoregional invasive gastric adenocarcinoma from 2004 to 2013 were included. A standard surgical resection was performed for all the patients with (*n* = 16) or without (*n* = 433) neoadjuvant treatment.

**Results:**

In this study, 301 men and 148 women with a median age of 58 (range 21–88) years were included. The median total numbers of examined lymph nodes were 9 (range 0–55). Ninety-five patients (21.2%) had adequate (≥ 16) lymph node examination, and 70 patients (15.6%) had no examined lymph nodes. In univariate analysis, total or near total gastrectomy (*P* <  0.001), advanced node stage (*P* < 0.001), primary tumor size > 6 cm (*P* < 0.001), and the presence of perineural invasion (*P* = 0.039) were associated with more average number of examined lymph nodes. On multivariate analysis, node stage (*P* < 0.001) and type of surgery (*P* = 0.008) were independent predictive factors.

**Conclusion:**

In this study, approximately one in five patients with gastric adenocarcinoma had sufficient lymph node assessment. More studies are suggested for identifying a true inadequate lymph node dissection from insufficient lymph node assessment.

## Highlights

1. In this study, the median total numbers of examined lymph nodes were 9 (range 0–55).
2. Ninety-five patients (21.2%) had adequate (≥ 16) lymph nodes examination, and 70 patients (15.6%) had no examined lymph nodes.
3. Total or near total gastrectomy, advanced primary tumor and node stage, tumor size > 6 cm, and the presence of perineural invasion were associated with more median number of examined lymph nodes.
4. Advanced node stage and total or near total gastrectomy were independent predictive factors for adequate lymph node assessment.

## Background

Gastric cancer remains the fourth most frequent cancer and the second leading cause of cancer death worldwide. Despite a decline in incidence of gastric cancer in the western countries, it is still a major malignant disease [1].

In most countries, this malignancy present at late stage due to undefined risk factors and non-specific symptoms. Surgery is the mainstay of curative treatment of gastric cancer. In gastric cancer surgery, no residual tumor (R0) resection is an ultimate goal; however, there has been strong argue regarding the degree of lymph node (LN) dissection. This argument involves sufficient surgical and pathological staging and satisfactory adjuvant therapy. Generally, limited LN dissection (D1) involves perigastric LNs surrounded by 3 cm from the primary tumor, extended LN dissection (D2) extends the dissection outside D1 to include LNs surrounding the hepatic and splenic arteries, and superextended LN dissection (D3) further includes LNs in the retropancreatic, paraaortic, and the root of the mesocolon LNs. Further LN dissection such as D2 resection may potentially offer more precise pathologic staging, enhanced regional tumor control, and potential survival improvement. All these issues, however, need to be proven, because the results of randomized clinical trials have failed to confirm a clear disease control and survival benefit to date [2].

In the fifth edition of the American Joint Committee on Cancer (AJCC) Tumor, Node, Metastasis (TNM) classification for gastric cancer, the number of positive nodes was considered as the base of LN classification. This classification showed superiority over the prior classification system in terms of LN stage as a prognostic factor. However, as it is expected, the more LN evaluated, nodal metastases detection is more likely. The number of nodal examined depends not only on the extent of LN dissection, but also on the lymph node retrieval. Therefore, when the number of examined LNs is insufficient for diagnosis, the nodal stage may be underestimated, which is so-called stage migration [3].

The 7th edition of the AJCC staging manual (2010) revised the nodal classification system such that N1 = 1–2 positive LNs; N2 = 3–6 positive LNs; N3a = 7–15 positive LNs; and N3b > 15 LNs. As such, the AJCC now recommends that at least 16 LNs be assessed per patient. The staging changes attempt to minimize the impact of surgical dissection on gastric cancer staging and to improve the prognostic ability of N-staging compared to that in the 5th/6th editions. Unfortunately, despite the changes to simplify staging, the number of LNs assessed in each gastric cancer case varies, and in many cases, the number reported per specimen is less than current recommendations [3].

The present study aimed to investigate the sufficient (≥ 16) lymph node evaluation in patients with resected gastric adenocarcinoma, and review of the literature.

## Methods

In this retrospective study, a chart review was performed on 449 patients with resected locoregional invasive gastric adenocarcinoma who were treated and followed up at a referral academic hospital, between 2004 and 2013. A minimum sample size required for the study was calculated based on the value of standard deviation of the mean total number of assessed LN (SD = 11.02) in previous study by Zhao [4]. Accordingly, a minimum of 117 patients were estimated for a precision of 2%. Exclusion criteria in this study were in situ or metastatic tumor, pathologies other than adenocarcinoma, and unresectable or inoperable gastric cancer. Additionally, patients who had been treated with palliative surgery were excluded. The patients’ cancers were reclassified according to the 8th edition of the AJCC. Four hundred and thirty-three patients underwent primary surgery, and the remaining 16 cases received neoadjuvant chemotherapy and/or chemoradiation before curative surgical resection. Initial investigation involved a history taking and physical examination, upper GI endoscopy, laboratory test, and chest, abdominal, and pelvic computed tomography (CT) scans.

All statistical analysis was performed using IBM SPSS 22. The median, as well as the percentage of patients with sufficient (≥ 16) and insufficient (< 16) evaluated LN, was initially calculated. All potential clinical (age, sex, tumor location, type of surgery) and pathological (histologic form, tumor stage, node stage, tumor grade, lymphovascular invasion, perineural invasion, tumor size, surgical margin status, and the total and adequate assessed LNs) variables were analyzed. The outcome variable were measured based on the median and adequate (≥ 16) total number of assessed lymph node. The impact of all potential variables on the median and adequate (≥ 16) total number of assessed lymph node was analyzed using non-parametric (Mann-Whitney *U* test and Kruskal-Wallis test) tests respectively. Initially, a univariate analysis for the dependent variable adequate (≥ 16) LN assessment was performed. In the final step, all significant factors were included in a stepwise multivariate logistic analysis. All statistical tests were two-ended, and *p* values less than 0.05 were considered statistically significant.

## Results

**Fig. 1:**
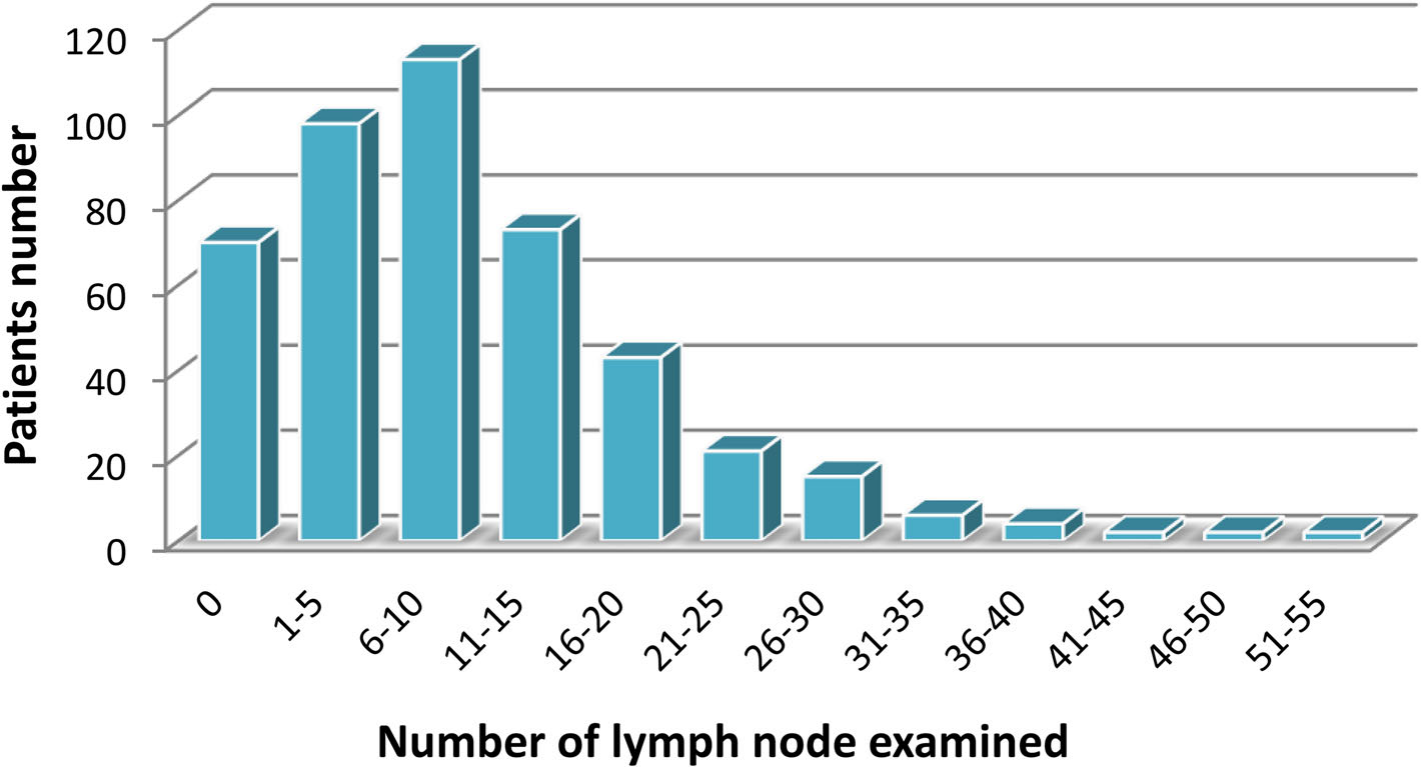
Relative distribution of total lymph node evaluation in 449 patients with resected gastric cancer

In this study, 301 men and 148 women with a median age of 60 (range 21–88) years were included. Two hundred and twenty-one patients were less than 60 years old, and 228 patients were older than or equal to 60 years old. The distribution of histologic form (*P* < 0.001), tumor grade (*P* = 0.027), and tumor size (*P* = 0.016) was significantly different among tumor location (Table 1). Accordingly, diffuse gastric involvement tended to be presented with larger tumor size, to have higher rate of poorly differentiated tumor and diffuse histologic type. The median total numbers of evaluated lymph nodes were 9 (range 0–55). Only 95 patients (21.2%) had equal or more than 16 lymph nodes evaluation, and 70 patients (15.6%) had no any lymph nodes for evaluation. Relative distribution of total lymph node evaluation has been illustrated in Fig. 1. Two hundred and sixty-two patients (58.3%) were node positive. Association of potential variables on median total lymph node count has been illustrated in Table 2. Additionally, an association was found between positive node and lymphatic vascular invasion (*P* < 0.001), the presence of perineural invasion (*P* < 0.001), and advanced T stages (*P* < 0.001). In univariate analysis, type of gastrectomy (*P* < 0.001, node stage (*P* < 0.001), primary tumor size (*P* < 0.001), and perineural invasion (*P* = 0.039) were significant variables (Table 3). In multivariate analysis using logistic regression method, N3 node stage (*P* < 0.001, OR = 5.907, CI = 3.462–10.081) and total or near total gastrectomy (*P* = 0.008, OR = 2.146, CI = 1.221–3.772) were independent variables (Table 4).

**Table 1:** Patient and tumor characteristics by tumor location

Characteristics	Tumor location (%)				*P* value
	Proximal	Distal	Diffuse	Total	
Gender					0.298
Male	124 (42.8)	137 (47.2)	29 (10)	290 (100)	
Female	50 (35.0)	77 (53.8)	16 (11.2)	143 (100)	
Age					0.463
Median (range)	60 (27–84)	58 (21–82)	59 (26–84)	60 (21–88)	
Histologic form					< 0.001
Diffuse type	68 (39.8)	73 (42.7)	30 (17.5)	171 (100)	
Intestinal type	96 (41.9)	122 (53.3)	11 (4.8)	229 (100)	
T stage					0.250
1	4 (21.1)	13 (68.4)	2 (10.5)	19 (100)	
2	21 (44.7)	25 (53.2)	1 (2.1)	47 (100)	
3	103 (40.9)	122 (48.4)	27 (10.7)	252 (100)	
4	32 (36.8)	43 (49.4)	12 (13.8)	87 (100)	
N stage					0.641
No	49 (45.8)	47 (43.9)	11 (10.3)	107 (100)	
N1	32 (45.0)	30 (42.3)	9 (12.7)	71 (100)	
N2	36 (36.4)	54 (54.5)	9 (9.1)	99 (100)	
N3	37 (41.1)	40 (44.5)	13 (14.4)	90 (100)	
Tumor grade					0.027
Well differentiated	42 (47.7)	43 (48.9)	3 (3.4)	88 (100)	
Moderately differentiated	59 (41.6)	74 (52.1)	9 (6.3)	142 (100)	
Poorly differentiated	69 (36.9)	92 (49.2)	26 (13.9)	187 (100)	
Lymphatic-vascular invasion					0.055
Negative	52 (46.4)	54 (48.2)	6 (5.4)	112 (100)	
Positive	110 (37.0)	150 (50.5)	37 (12.5)	297 (100)	
Perineural invasion					0.388
Negative	61 (43.6)	67 (47.8)	12 (8.6)	140 (100)	
Positive	100 (37.3)	137 (51.1)	31 (11.6)	268 (100)	
Tumor size					0.016
≤ 6 cm	101 (38.4)	142 (54.0)	20 (7.6)	263 (100)	
> 6 cm	56 (45.2)	50 (40.3)	18 (14.5)	124 (100)	
Surgical margin status					0.292
Free	113 (38.4)	152 (51.7)	29 (9.9)	295 (100)	
Involved	47 (44.8)	45 (42.8)	13 (12.4)	105 (100)	
Total LN examined					0.099
Median (range)	9 (0–48)	7 (0–55)	11 (0–45)	8 (0–55)	
Adequate LN examined					0.092
Inadequate (< 16 LNs)	131 (38.5)	177 (52.1)	32 (9.4)	340 (100)	
Adequate (≥ 16 LNs)	43 (46.2)	37 (39.8)	13 (14.0)	93 (100)	

*LN* lymph node

**Table 2:** Association of potential variables on median total lymph node count in 449 patients with resected gastric adenocarcinoma

Variables	Patients’ no.	Median total LNs examined (Q1, Q3)	*P* value
Patients’ sex			
Male	301	8 (3, 14)	
Female	148	8 (3, 13)	0.964
Patients’ age			
< 60 years	221	8 (3, 12)	
≥ 60 years	228	8 (3, 15)	0.387
Primary tumor site			
Diffuse	45	11 (5, 18)	
Non diffuse	388	7 (2, 12)	0.298
Type of gastric surgery			
Total or near total gastrectomy	246	10 (5, 17)	
Partial or subtotal gastrectomy	167	7 (3, 11)	0.001
Primary tumor stage			
T1–2	69	7 (4, 11)	
T3–4	348	9 (4, 15)	0.024
Node stage			
N0–2	286	7 (4, 12)	
N3	92	17 (11, 25)	< 0.001
Tumor size			
≤ 6 cm	263	7.5 (4, 12)	
> 6 cm	124	12 (6, 19)	< 0.001
Neoadjuvant treatment			
Not received	433	8 (4, 14)	
Received	16	5 (0, 6)	0.132
Surgical margin status			
Free	295	8 (4, 14)	
Involved	105	9 (5, 16)	0.445
Tumor grade			
Grades I–II	234	8 (3, 14)	
Grade III	198	8 (4, 13)	0.567
Lymphatic-vascular invasion			
Negative	112	8 (4, 13)	
Positive	297	8 (4, 14)	0.590
Perineural invasion			
Negative	140	7 (4, 12)	
Positive	268	9 (4, 16)	0.039
Histologic form			
Diffuse type	171	8 (4, 15)	
Intestinal type	229	8 (3, 13)	0.372

*LN* lymph node, *Q1* quartiles 1 (percentile 25), *Q3* quartiles 3 (percentile 75)

**Table 3:** Univariate analyses for the dependent variable, adequate (≥ 16) LN assessment in 449 patients with resected gastric adenocarcinoma

Variables	Patients’ no.	No. of adequate (≥ 16) LN assessment (%)	*P* value
Patients’ sex			
Male	301	64 (21.3)	
Female	148	31 (20.9)	0.938
Patients’ age			
< 60 years	221	51 (21.3)	
≥ 60 years	228	44 (19.3)	0.327
Primary tumor site			
Diffuse	45	13 (28.9)	
Non diffuse	388	80 (20.6)	0.201
Type of gastric surgery			
Total or near total gastrectomy	246	72 (21.9)	
Partial or subtotal gastrectomy	167	23 (13.8)	< 0.001
Primary tumor stage			
T1–2	69	12 (17.4)	
T3–4	348	83 (23.9)	0.243
Node stage			
N0–2	286	43 (15.0)	
N3	92	51 (55.4)	< 0.001
Tumor size			
≤ 6 cm	263	48 (17.3)	
> 6 cm	124	45 (35.2)	< 0.001
Neoadjuvant treatment			
Not received	433	94 (22.0)	
Received	16	1 (6.3)	0.087
Surgical margin status			
Free	305	66 (21.6)	
Involved	107	27 (25.2)	0.444
Tumor grade			
Grades I–II	234	49 (20.9)	
Grade III	198	46 (23.2)	0.567
Lymphatic-vascular invasion			
Negative	117	24 (20.5)	
Positive	305	70 (23.0)	0.590
Perineural invasion			
Negative	145	24 (16.6)	
Positive	276	70 (25.4)	0.039
Histologic form			
Diffuse type	182	44 (24.2)	
Intestinal type	234	48 (20.5)	0.372

*LN* lymph node

**Table 4:** Independent variables associated with adequate (≥ 16) lymph node assessment in resected gastric adenocarcinoma

Variables	*P* value	Odds ratio	CI (95%)
Type of gastric surgery			
Partial or subtotal gastrectomy			
Total or near total gastrectomy	0.008	2.146	1.221–3.772
Node stage			
N0–2			
N3	< 0.001	5.907	3.462–10.081
Tumor size			
≤ 6 cm			
> 6 cm	0.120	1.535	0.894–2.635
Perineural invasion			
Negative			
Positive	0.674	1.138	0.624–2.075

*CI* confidence interval

## Discussion

Gastric cancer remains a major health problem not only in developing, but also in developed countries. Surgical treatment including complete resection of primary tumor and regional LN dissection plays an essential role in treating these patients. Theoretically, an insufficient LN dissection increases the risk of potential microscopic and gross residual tumor cell, higher rate of recurrent disease, and poorer prognosis [5]. It is believed that by increasing the number of surgically dissected and pathologically harvested LNs, surgical and pathological staging will be more accurate. Subsequently, this can potentially enhance locoregional tumor control and improves oncologic outcomes following gastric cancer surgery. Accordingly, a minimum number of 16-LN evaluation was recommended to achieve precise staging [6]. Despite many years after changing in gastric cancer staging, only 33% of patients had an adequate lymph node assessment.

The occurrence of gastric cancer in patients younger than 40 years old is uncommon; however, after which, its incidence increases progressively. In the current study, the patients with a median age of 60 years old were younger than that of the results of previous reports in which the average median age of 27,214 patients in 15 report series was 65.5 (range 54–71) years old [1, 4, 6–18] (Table 5). During recent decades, the frequency of primary tumor location has been changed in favor of proximal gastric cancers. At present, proximal gastric cancers including gastroesophageal junction lesions are diagnosed more frequently than in the past. Nevertheless, most gastric cancers still originate from distal stomach. The largest percentage of gastric cancers still arise within the antrum or distal stomach [19]. In this study, most of the lesions were in the distal of stomach (50.5%). In major reported series, the average total number of lymph node evaluated was 27.7 (range 8.4–35.3) for 7643 patients in 8 studies [4, 6, 8–12, 20]. In this research, the average total number of evaluated lymph node was 11.1. Furthermore, in the current study, 58% of all patients had stage III which was higher than that of major reported series in which this value was 31.2% (range 15.5%–64%) for 22,994 patients in 10 series [2, 7–10, 13–16, 18, 20]. In gastric cancer, insufficient lymph node assessment is a common finding in the literature. By analyzing data of 15 series including 27,942 patients, 52.2% (range 17.6–94.2%) of all patients had adequate (≥ 16) lymph node evaluation [1, 2, 4, 6–9, 13–18, 21, 22] (Table 5). In the present study, only 21.2% of patients had a sufficient lymph node assessment which seems to be much lower than that of average value in the literature.

**Table 5:** The status of lymph nodes assessment in resected gastric adenocarcinoma in major reported series in the literature

Authors	No. of patients	Stage	Median age	Mean TLN	Median TLNE	Mean % of stage III	% of ALNE
Biffi et al. [1]	114	I–II	63	NR	22	NR	78.9
Bouvier et al. [6]	749	I–III	68	8.4	NR	NR	17.6
Bruno et al. [10]	367	I–IV	67	17.4	15	32.6	NR
Chen et al. [7]	1101	I–IV	58	NR	NR	35.8	68.5
Coburn et al. [14]	10,807	I–IV	70	NR	9	27.5	29
Deng et al. [12]	196	II–IV	69	15.7	NR	NR	75
Gholami et al. [18]	742	I–III	65	NR	NR	15.5	65
Giuliani et al. [11]	154	I–III	65	22.6	NR	NR	NR
Huang et al. [8]	634	I–IV	NR	NR	NR	44.2	83.1
Huang et al. [22]	236	I–IV	58	23.8	23	NR	84.7
Ichikura et al. [9]	925	I–IV	57	32	30	16.4	77
Lee et al. [13]	4789	I–IV	54	31.9	30	28	94.2
Marubini et al. [2]	615	I–IV	NR	NR	NR	39.8	73.2
Schwarz and Smith [15]	1377	II–III	68	NR	17	48.4	74.7
Shen et al. [16]	1637	I–IV	65	NR	19	54.7	81.4
Smith et al. [21]	3793	I–II	71	NR	8	NR	25
Zhao et al. [4]	227	I	57	18.51	NR	NR	55.5
Present study	449	I–III	60	11.1	9	58.3	21.2
Total	28,912	I–V	65.4	27.7	15.1	31.7	51.7

The cause of insufficient lymph node assessment is multifactorial. A cooperation between the surgeon for doing optimal lymph node dissection and pathologist for doing sufficient lymph node assessment is required [23]. However, according to multiple studies, a variety of factors can affect both parties, including ethic background such as body mass index (that affect the surgeon’s ability to perform an adequate lymphadenectomy), age (younger patients are more likely to have an adequate LN assessment), and region in which the surgery is performed [21]. In this study, age and sex have no significant relation with examined lymph nodes. Regarding the tumor and treatment factors, the average number of evaluated lymph node was associated significantly with primary site, node stage, tumor size, perineural invasion, type of surgery, and neoadjuvant treatment. Higher lymph node examined was yield in total or near total gastrectomy, stage N3, tumor size > 6 cm, and presence of perineural invasion. The type of gastric surgery and extent of LN dissection remains an important factor in the adequacy of lymph node sampling and survival outcomes. Therefore, the surgeon can be considered as one of the most independent predictive factor in gastric cancer for achieving adequate lymph node sampling, R0 resection and locoregional disease control [24]. In this study, total gastrectomy was associated with more average number of examined lymph nodes compared to those with subtotal gastrectomy. In the current study, due to large number of surgeons, pathologists, and hospitals, to investigate any association between adequate lymph node assessment and the surgeon, pathologists, and hospitals was not possible. Many factors affected the probability of sufficient LN assessment in the patients. In the final multivariable Cox model, factors of age, sex, year of diagnosis, type of surgery, tumor stage, and tumor grade remained significant predictors for adequate lymph node assessment [2]. There are conflicting reports regarding the impact of different variables on examined lymph nodes. Gholami et al. analyzed 742 patients who underwent gastrectomy for gastric adenocarcinoma. They found patients with more advanced T and N stage, younger age, and D2 lymphadenectomy tended to have adequate LN assessment. The rest of the variables including resection margin status, type of gastrectomy, grade of tumor, and sex were not related to obtain adequate lymph node dissection [18].

Biffi et al. demonstrated no association between the factors of sex, age, type of adenocarcinoma, T stage, and grade of the tumor and the number of dissected lymph nodes. There were dissected lymph nodes > 15 only in patients that did not receive neoadjuvant treatment [1].

Zhao et al. reported that no relation was noted between age, tumor site, or tumor grade and number of dissected lymph nodes. More women were in the ≤ 15 LN group than in those with > 15 LN [4].

Huang et al. was performed the study in 634 patients with gastric cancer. In their study, the clinicopathological characteristics of patients like gender, age, tumor size, tumor location, grade of tumor, and T stage did not influence the number of examined lymph nodes [19]. Many reports showed a major role for surgical volume and experience of the surgeons and pathologist on lymph node retrieval in gastric cancer. High volume of surgery such as total and near total gastrectomy provides more removal and examination of regional LNs. In addition, in cases with higher T stage, gross LN involvement is more frequent which facilitates more LN detection and dissection by the surgeon and more LN retrieval by pathologist. Likewise, neoadjuvant therapies such as chemotherapy and chemoradiation can potentially shrinkage and disappear gross involved LNs and subsequently decrease the number of LN detection, dissection, and examination in gastric cancer. Conversely, in patients with early-stage gastric cancer, low-volume limited surgery may be associated with inadequate LN staging in these patients [17, 25].

In Iran, many factors including the paucity of experienced surgical oncologist and pathologist and particularly infrequent extended LN dissection may contribute to insufficient LN assessment in gastric cancer.

The limitation of the current study were retrospective chart review of the patients’ medical records, unknown operative details regarding surgical approach and LN location, non-uniform pathologic reports, and relatively small sample size of a single institution data based.

## Conclusion

This study indicates that only one fifth of patients with gastric adenocarcinoma underwent sufficient lymph nodes assessment in Shiraz, Iran. As well, in this research, about 15% of the patients had no lymph nodes for assessment. Multicenter studies with larger sample size are suggested to confirm these results and to identify a true insufficient lymph node dissection from insufficient lymph node detection.

## Data Availability

The datasets generated and analyzed during the current study are not publicly available due to our department privacy, but are available from the corresponding author on reasonable request.

## Abbreviations

AJCC: American Joint Committee on Cancer
CT: Computed tomography
LN: Lymph node
N stage: Node stage
SD: Standard deviation
T stage: Tumor stage
TNM: Tumor, Node, Metastasis
